# Development and approval of live attenuated influenza vaccines based on Russian master donor viruses: Process challenges and success stories

**DOI:** 10.1016/j.vaccine.2016.08.018

**Published:** 2016-10-26

**Authors:** Larisa Rudenko, Leena Yeolekar, Irina Kiseleva, Irina Isakova-Sivak

**Affiliations:** a Department of Virology, Institute of Experimental Medicine, 12 Acad. Pavlov Street, Saint Petersburg 197376, Russia; b Vaccine Production, Serum Institute of India, 212/2 Hadapsar, Pune, India

**Keywords:** Influenza, Live attenuated influenza vaccine, Seasonal vaccine, Potentially pandemic viruses, Preclinical studies, Clinical trials, Pandemic preparedness, Technology transfer

## Abstract

Influenza is a viral infection that affects much of the global population each year. Vaccination remains the most effective tool for preventing the disease. Live attenuated influenza vaccine (LAIV) has been used since the 1950s to protect humans against seasonal influenza. LAIVs developed by the Institute of Experimental Medicine (IEM), Saint Petersburg, Russia, have been successfully used in Russia since 1987.

In 2006, the World Health Organization (WHO) announced a Global action plan for influenza vaccines (GAP). WHO, recognizing potential advantages of LAIV over the inactivated influenza vaccine in a pandemic situation, included LAIV in the GAP.

BioDiem Ltd., a vaccine development company based in Melbourne, Australia which held the rights for the Russian LAIV, licensed this technology to WHO in 2009. WHO was permitted to grant sub-licenses to vaccine manufacturers in newly industrialized and developing countries to use the Russian LAIV for the development, manufacture, use and sale of pandemic and seasonal LAIVs. To date, WHO has granted sub-licenses to vaccine manufacturers in China (Changchun BCHT Biotechnology Co., Ltd.), India (Serum Institute of India Pvt. Ltd.) and Thailand (Government Pharmaceutical Organization). In parallel, in 2009, IEM signed an agreement with WHO, under which IEM committed to supply pandemic and seasonal candidate vaccine viruses to the sub-licensees.

This paper describes the progress made by collaborators from China, India, Russia and Thailand in developing preventive measures, including LAIV against pandemic influenza.

## Highlights

• LAIV has been used in Russia for decades.
• Russian LAIV consistently provides superior effective protection against influenza.
• It was incorporated into the WHO global pandemic influenza action plan.
• A number of Russian LAIVs against pandemic influenza viruses have been prepared.
• Russian LAIV technology was transferred to a number of developing countries.

## 1 Introduction

Influenza is a life-threatening viral infection that may affect up to 40% of the world’s population each year [1]. Vaccination remains the most effective means of preventing seasonal influenza epidemics, which are associated with significant morbidity and mortality worldwide.

Long-term observations have shown that live attenuated influenza vaccine (LAIV) has some major advantages over inactivated influenza vaccine (IIV). These advantages include ease of needle-free delivery, extremely low rate of adverse reactions, smaller infrastructure requirement for manufacturing, limited downstream processing and significantly higher yield in eggs (nearly 15 doses of LAIV can be produced from one embryonated egg). These factors make LAIV especially attractive for developing countries with a large population. Furthermore, the concept of replicating the vaccine virus in the nasal cavity and thus generating a specific immune response at the site of infection appears to be the most appropriate mode of immunization.

All these features of LAIV become even more relevant with the emergence of potentially pandemic influenza viruses of different serotypes. The World Health Organization (WHO) recognized the advantages of LAIV over IIV in the event of a pandemic and therefore included LAIV in its Global action plan for influenza vaccines [2], [3].

In Russia, LAIV has a long history of development, stage-wise improvement, licensing and use in public health. Since 1987, Russian LAIV has been used for the prophylaxis of influenza in children aged over three years, in adults and in the elderly. Currently, reassortant viruses for Russian LAIV are prepared by classical reassortment in eggs of wild-type influenza A and B viruses with two cold-adapted master donor viruses (MDVs) as a backbone: A/Leningrad/134/17/57 (H2N2) and B/USSR/60/69, respectively. Russian LAIV has been shown through studies to consistently provide superior effective protection, especially in children, compared to IIV.

The other LAIV used in the world, based on Ann-Arbor backbone has likewise been shown to provide superior protective efficacy in children, compared to IIV, through randomized controlled trials. Some recent studies have however suggested that since 2011 there has been a reduction in the comparative efficacy of the Ann-Arbor based LAIV compared to IIV. The cause is still unknown however efforts to understand this sudden loss of efficacy are focusing on the role of the A/California H1N1 component as well as the inclusion of a second B-strain, and it is expected that this issue will be successfully addressed.

## 2 Transfer of Russian LAIV technology

BioDiem Ltd. (Melbourne, Australia), holding the rights for use of the Russian MDVs, licensed Russian LAIV technology to WHO [4]. The agreement with BioDiem permitted WHO to grant sub-licenses to vaccine manufacturers in the newly industrialized countries (NICs) and developing countries within the framework of the WHO influenza vaccine technology transfer project. Since 2009, WHO has signed agreements with the Changchun BCHT Biotechnology Co., Ltd. (BCHT, Changchun, Jilin, China), the Serum Institute of India Pvt. Ltd. (SIIPL, Pune, India) and the Government Pharmaceutical Organization (GPO, Bangkok, Thailand) for the development, manufacture, use and sale of the egg-based LAIV using Russian MDVs.

At the same time, the Institute of Experimental Medicine (IEM), Saint Petersburg, Russia – the sole developer of reassortant strains for Russian LAIV – signed an agreement with WHO. Under this agreement, there were two main areas of work: development of seasonal LAIV candidates according to biannual WHO recommendations for influenza vaccine compositions, and Development of LAIV candidates against potentially pandemic influenza viruses.

During the period 2009–2015, IEM developed and transferred to WHO nine seed-LAIVs for seasonal vaccines and one H1N1 pandemic seed-LAIV for further distribution to manufacturers (Table 1, Table 2). All of these LAIV candidates were accompanied by strain certificates drawn up in accordance with international standards, which included detailed descriptions of the seed-LAIV generation and the quality-control attributes; that is, antigenicity and identity tests, phenotypic and propagation characteristics, genetic stability data, full genome sequencing, sterility control and safety preclinical testing in laboratory animals.

In 2012, the increased international demand for the Russian LAIV prompted the establishment of an additional (back-up) laboratory facility at the Centers for Disease Control and Prevention (CDC), Atlanta, Georgia, United States of America (USA) for parallel preparation of the LAIV candidates based on Russian MDVs for international use. The organizations responsible for this were WHO, the Biomedical Advanced Research and Development Authority (BARDA), USA, and IEM. This decision made it possible to reduce the unpredictable risks associated with the production of LAIV candidates in one laboratory and in one country alone. The back-up laboratory has been working successfully [5], [6], [7], [8] and a number of reassortants have been used to produce the LAIV in India and China (Table 4).

## 3 Creation of a modern high-tech pathogenic agent’s laboratory facility and development of potentially pandemic LAIV candidates

To meet the demand for high-quality LAIV seed viruses for further distribution between dedicated manufacturers, it was necessary to reconstruct and build a new state-of-the-art facility at IEM to work with pathogens of biosafety level BSL-2 and BSL-3 groups, in compliance with all international biosafety standards. For this purpose WHO, in collaboration with BARDA, allocated all necessary funds, and the construction was completed in 2014. Since then, the facility has been fully operational and has been certified by the Russian Ministry of Health. This laboratory occupies a total area of 550 m^2^, with additional technical and engineering facilities covering an area of over 1000 m^2^. Additional financing for the purchase of the equipment was provided by the Program for Appropriate Technologies in Health (PATH), Seattle, Washington, USA.

Including the LAIV into the WHO Global action plan for influenza vaccines required the generation of a panel of LAIV candidates against potentially pandemic H5N1, H2N2, H7N9 and H7N3 influenza viruses. According to collaborative agreements between IEM, PATH and WHO, the following potentially pandemic LAIV candidates were developed: A/17/turkey/Turkey/05/133 (H5N2) [9], A/17/Vietnam/04/65107 (H5N2) [9], A/17/California/66/395 (H2N2) [10], A/17/Anhui/2013/61 (H7N9) [11], A/17/mallard/Netherlands/00/95 (H7N3) [12] (Table 2).

### 3.1 Preclinical testing

Studies on the safety, immunogenicity and protective efficacy of these potentially pandemic LAIV candidates in ferrets were conducted in collaboration with experts from the Centre for Infectious Disease Control (Bilthoven, Netherlands), University of Pittsburgh (Pittsburgh, Pennsylvania, USA), Southeast Poultry Research Laboratory (Athens, Georgia, USA), CDC (Atlanta, Georgia, USA) and ViroClinics Biosciences (Rotterdam, Netherlands) [9], [10], [11], [13], [14], [15], [16]. It was demonstrated that immunization with the potentially pandemic LAIV candidates induced a strong immune response, and the ferrets were protected against homologous and heterologous wild-type virus challenge. Replication of challenge viruses in the upper and lower respiratory tracts of immunized animals was significantly reduced compared to the controls, and no signs of disease were observed in any of the vaccinated animals (Table 3).

### 3.2 Clinical trials

Phase I clinical trials of the potentially pandemic LAIV candidates of H5N2, H7N3, H2N2 and H7N9 subtypes were conducted in clinics of the Research Institute of Influenza (Saint Petersburg, Russia). All trials were randomized, double-blinded, placebo-controlled studies in healthy adults. In order to minimize the potential for vaccine virus to release into the environment vaccinations took place in an inpatient isolation unit; subjects were tested for influenza virus in nasal swabs prior to vaccination; presence of vaccine virus in nasal swabs was assessed daily; subjects were to be kept in the isolation unit until shedding was no longer detected; an independent Safety Monitoring Committee reviewed the safety and shedding data.

These LAIVs were found to be safe, well tolerated and immunogenic in human adult volunteers [4], [17], [18], [19], [20]. Catarrhal symptoms, such as hyperemia and sore throat, were the most frequently reported adverse events with the pandemic LAIVs. The frequency of reporting sore throat following LAIV administration ranged from 11% (for H2N2 LAIV) to 27% (for H7N3 LAIV). The vaccine viruses were recovered from the nasal or throat swabs of vaccinees by virus culture in embryonated eggs, with most of the subjects scoring positive for virus shedding only on the first day after vaccination. No vaccine virus or viral RNA was detected in any of the placebo recipients after either the first or the second vaccine dose for any of the five LAIVs reported here. Importantly, the lack of vaccine virus transmission to placebo recipients was observed despite co-housing all subjects (vaccine and placebo recipients) in the same isolation facility. This finding may support the lack of vaccine transmissibility of LAIVs.

**Fig. 1:**
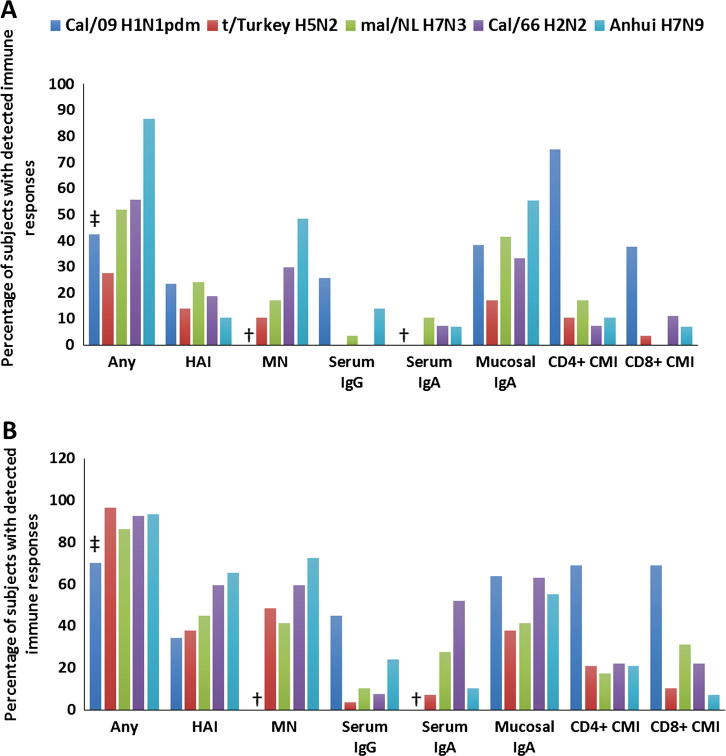
Antibody and cell-mediated immune responses to pandemic LAIVs in clinical trials. (A) Immune responses after dose 1; (B) Immune responses after two doses of the vaccine. Cal/09 H1N1pdm: A/17/California/2009/38 (H1N1); t/Turkey H5N2: A/17/turkey/Turkey/05/133 (H5N2) LAIV; mal/NL H7N3: A/17/mallard/Netherlands/00/84 (H7N3) LAIV; HAI – hemagglutination inhibition; MN – microneutralization; CMI – cell-mediated immune response. † MN and serum IgA tests were not performed for Cal/09 H1N1pdm LAIV. ‡ Only antibody immune responses are included in calculations because only a small proportion of subjects was tested for CMI.

Immunization with potentially pandemic LAIVs induced high levels of hemagglutination inhibition (HAI) and local secretory antibodies, as well as long-lasting B-cell immunological memory against antigenically related influenza virus. Taking into account all the assays conducted (HAI, microneutralization, enzyme-linked immunosorbent assay [ELISA] and cytokine flow cytometry), an immune response was observed in 70.2% of H1N1pdm09-LAIV recipients, 96.6% of H5N2-LAIV recipients, 86.2% of H7N3-LAIV recipients, 92.6% of H2N2-LAIV recipients and 93.1% of H7N9-LAIV recipients. In contrast, none of the placebo recipients exhibited any response (Fig. 1).

## 4 Progress of development of LAIV in the NICs

The declaration of 2009 H1N1 influenza pandemic prompted SIIPL and GPO to start immediate manufacturing of pandemic LAIV.

Using LAIV production technology adapted from IEM, GPO successfully produced H1N1pdm09 LAIV, conducted preclinical and clinical trials of this vaccine, and registered it in 2011 [21]. Since the registration of H1N1pdm09 LAIV, intensive studies of another pre-pandemic LAIV candidate – H5N2 – have been carried out at GPO. These studies are progressing well and are nearing completion. Thus, Thailand is well on the way to having LAIVs ready to respond to a pandemic.

In 2011, the BCHT signed an agreement with WHO, and since then the company has built a manufacturing plant and adopted the LAIV production technology. BCHT is currently conducting Phase I clinical trials of seasonal LAIV.

The LAIV promotion in India was the most successful. SIIPL developed a lyophilized monovalent H1N1pdm09 LAIV that was reconstituted with water for inhalation; it showed satisfactory stability of 9 months. Preclinical studies in ferrets showed remarkable protection after challenge with wild-type virus, with one dose of the vaccine [13].

Clinical experience with the monovalent H1N1pdm09 LAIV showed a good safety profile of the vaccine in all age groups, including children aged over 3 years. The immune response was in line with the other LAIVs. The vaccine was developed, licensed and commercialized within 12 months [22]. Post-marketing surveillance and the periodic safety update reports further established the safety of the vaccine [23]. A case-control study also demonstrated high effectiveness of the vaccine during the epidemic [24].

One of the major issues related to pandemic preparedness is the availability of a pre-existing manufacturing setup that can be geared up for large-scale vaccine production in the face of a pandemic. This would be possible only if continual manufacturing of the influenza vaccine for the annual vaccination was maintained. Understanding this need, SIIPL ventured into development of a seasonal trivalent LAIV, prepared using the same manufacturing process and stabilizer as the monovalent H1N1pdm09LAIV, and with a stability of 9 months.

The trivalent LAIV was tested in a ferret challenge study, and established protection from all three strains. The clinical trials involved safety assessment and immunogenicity testing in individuals aged over 2 years, which both demonstrated acceptable results. The vaccine was licensed by the Indian authorities in January 2014 and is prequalified by WHO.

Since the development of LAIV at SIIPL five different compositions recommended for the northern and southern hemisphere have been manufactured and used successfully (Table 4).

Two clinical trials to assess efficacy in children aged 2–5 years were conducted by PATH in Bangladesh and Senegal [25]. The vaccine was found to be efficacious in this age group in Bangladesh. Surprisingly, the Senegal study indicated no protection in the same age group using the same lot of the vaccine. These contrasting findings are difficult to explain and need extensive analysis on antigenic make up of the circulating strains, population behavior and so on.

One more clinical trial of seasonal LAIV by the CDC is ongoing in children at Ballabgarh, Haryana, India (see vaccine composition in Table 4). This trial is in its second year of immunization and is expected to be completed by mid 2017. Furthermore, an age de-escalation study aimed at immunizing children aged 6–24 months is under consideration.

Some manufacturers are now producing quadrivalent vaccines to include both lineages of influenza type B which are co-circulating. Twice per year WHO recommends the composition of strains for both trivalent and quadrivalent vaccines. SIIPL developed a good manufacturing practice (GMP)-grade egg-based quadrivalent preparation of LAIV that was tested in the ferret model for immunogenicity and efficacy.

Further efforts are being made to develop rapid up scaling capacity for the vaccine production during a pandemic. To make the delivery user-friendly, the quadrivalent vaccine has been developed as a ready-to-use liquid vaccine and the nasal delivery devices have been upgraded.

Considering the unpredictable demand for influenza vaccines in India, and worldwide in general, pre-ordering for large quantity of eggs is a major challenge. An obvious solution to this problem is the development of a tissue culture-based LAIV. A well-characterized Madin-Darby canine kidney (MDCK) cell bank has been prepared by SIIPL and experimental batches have been prepared. Further work to develop GMP LAIV lots is underway.

The SIIPL is aiming towards a MDCK-based quadrivalent liquid LAIV that will have an ideal influenza vaccine profile including safety, efficacy, stability and ease of administration, as well as high capacity to rapidly increase the production.

## 5 Summary

1. Studies of LAIV in developing countries are based on over 40 years of monitoring the implementation of LAIV in Russia. A number of seasonal LAIV candidates using the A/Leningrad/134/17/57 (H2N2) and B/USSR/60/69 backbone were developed by IEM and the newly formed back-up laboratory at the CDC. All these vaccine strains were supplied to three countries: China (BCHT), India (SIIPL) and Thailand (GPO) for the production of LAIV and the preparation for vaccine registration.
2. A modern high-tech BSL-2 and BSL-3 facility was built in IEM with financial support from BARDA and WHO. Since 2009, a number of LAIV candidates against the most potentially pandemic influenza viruses subtypes – namely H1N1, H5N2, H7N3, H2N2 and H7N9 – have been prepared. Safety, immunogenicity and efficacy of these candidates in the animal models were demonstrated in collaboration with the Centre for Infectious Disease Control (Netherlands), University of Pittsburgh (USA), Southeast Poultry Research Laboratory (USA), CDC (USA) and ViroClinics Biosciences (Netherlands).
3. All the above-mentioned pandemic and pre-pandemic LAIV candidates have been included in the Phase I clinical trials in adult volunteers, in collaboration with PATH, the Research Institute of Influenza (Saint Petersburg, Russia) and Microgen (Moscow, Russia). Vaccines demonstrated a good safety profile and were well tolerated. The two-dose immunization schedule resulted in measurable serum and local antibody production, and generation of CD4^+^ and CD8^+^ memory T cells. The results of the clinical trials were published [4], [17], [18], [19], [20].
4. In 2009, GPO produced H1N1pdm LAIV, conducted preclinical and clinical trials, and registered the vaccine in Thailand. The work for preparation and registration of H5N2 pre-pandemic vaccine is in progress.
5. BCHT completed the construction of the manufacturing plant and produced vaccine clinical lots. Clinical trials began in March 2016. In 2019, BCHT intends to finalize registration of LAIV and begin its use for influenza prevention.
6. SIIPL registered the monovalent pandemic H1N1 LAIV in 2010 and the trivalent seasonal LAIVs in 2014 in India. Both the vaccines have been prequalified by WHO. Currently, Indian LAIV is used for the prophylaxis of seasonal influenza. Today, SIIPL is conducting research on the improvement of vaccine preparation and expansion of the age groups, and is aiming to produce MDCK-derived liquid LAIV.
7. The incorporation of production of LAIVs based on A/Leningrad/134/17/57 (H2N2) and B/USSR/60/69 donors of attenuation developed in Russia into the WHO global pandemic influenza action plan has led to significant progress on the development and the promotion of the LAIV for influenza prophylaxis in countries with large populations.

## Conflict of interest

All the authors have declared that have no conflict of interest, except for LeenaYeolekar who is an employee of SIIPL.

## Financial disclosure

The studies were supported by PATH and WHO.

## Figures and Tables

**Table 1:** List of seasonal LAIVs prepared by IEM on A/Leningrad/134/17/57 (H2N2) and B/USSR/60/69 backbone, which were transferred to WHO.

Vaccine strain designation (LAIV candidate)	Wild-type parental strain	Influenza season^a^
B/56/Brisbane/60/2008	B/Brisbane/60/2008 (Victoria lineage)	2009–2010
		2010–2011
		2011–2012
		2016–2017

A/17/California/2009/38 (H1N1)pdm09^b^	A/California/07/2009 (H1N1pdm)	2009–2010
		2010–2011
		2011–2012
		2012–2013
		2013–2014
		2014–2015

A/17/Perth/2009/87 (H3N2)	A/Perth/16/2009 (H3N2)	2010–2011
		2011–2012

B/60/Wisconsin/2010/125	B/Wisconsin/1/2010 (Yamagata lineage)	2012–2013

A/17/Victoria/2011/89 (H3N2)	A/Victoria/361/2011 (H3N2)	2012–2013
		2013–2014

A/17/Texas/2012/30 (H3N2)	A/Texas/50/2012 (H3N2)	2013–2014
		2014–2015

B/60/Massachusetts/2012/10	B/Massachusetts/2/2012 (Yamagata lineage)	2013–2014
		2014–2015

B/60/Phuket/2013/26	B/Phuket/3073/2013 (Yamagata lineage)	2015–2016

A/17/Bolivia/2013/6585 (H1N1)pdm09	A/Bolivia/559/2013 (H1N1)pdm2009	2015–2015

A/17/Hong Kong/2014/8296 (H3N2)	A/Hong Kong/4801/2014 (H3N2)	2016–2017

IEM – Institute of Experimental Medicine; LAIV – live attenuated influenza vaccine; WHO – World Health Organization.

a Northern hemisphere influenza season.

b Vaccine is registered in Russia.

**Table 2:** List of potentially pandemic LAIVs prepared by IEM on A/Leningrad/134/17/57 (H2N2) backbone, which were transferred to WHO.

Vaccine strain designation (LAIV candidate)	Wild-type strain	The stage of the study	Ref.
A/17/turkey/Turkey/05/133 (H5N2)	NIBRG–23 (H5N1), clade 2.2	Phase I clinical trial completed	[7], [19]
A/17/Vietnam/04/65107 (H5N2)	IDCDC–RG1 (H5N1), clade 1	Preclinical trials completed	[7]
A/17/California/66/395 (H2N2)	A/California/1/66 (H2N2)	Phase I clinical trial completed	[8], [18]
A/17/Anhui/2013/61 (H7N9)	A/Anhui/1/2013 (H7N9)	Phase I clinical trial completed	[9], [20]
A/17/mallard/Netherlands/00/95 (H7N3)	A/mallard/Netherlands/12/2000 (H7N3)	Phase I clinical trial completed	[10], [17]

IEM – Institute of Experimental Medicine; LAIV – live attenuated influenza vaccine; WHO – World Health Organization.

**Table 3:** Preclinical evaluation of pandemic LAIV candidates in animal models.

Pandemic/potentially pandemic LAIV candidate	Animal model	Main findings	Ref.
A/17/California/2009/38 (H1N1)pdm09	Ferrets	Single immunization induced high serum HAI antibody titers and the animals were protected against intratracheal wild-type pH1N1 virus challenge: virus replication in URT and LRT was reduced and no disease signs or severe broncho-interstitial pneumonia were observed in any of the vaccinated ferrets	[11]
A/17/turkey/Turkey/05/133 (H5N2)	Ferrets	Two doses elicited high levels of homologous and heterologous HAI antibody titers to clades 1, 2.1 and 2.2 H5N1 HPAI viruses. All vaccinated animals were fully protected against lethal challenge with homologous HPAI virus: no virus was detected in LRT, and the titers were significantly reduced in URT	[7]
VN1203/H5N1 rg	Mice	Two doses induced high titers of HAI antibodies which cross-reacted with clade 2.2 HPAI virus; animals were fully protected against lethal challenge with homologous and heterologous HPAI viruses	[12]
VN1203/H5N1 rg	Ferrets	Two doses elicited strong cross-reactive immune response; animals were protected from homologous and heterologous challenge with clade 1 and clade 2.2 HPAI viruses; a superior cross-protection of LAIV over inactivated vaccine was demonstrated in this challenge study	[13]
A/17/mallard/Netherlands/00/95 (H7N3)	Mice	Double immunization with high-dose vaccine elicited modest HAI antibody titers; nevertheless, the animals were protected against wild-type H7N3 virus replication in URT and LRT	[14]
A/17/mallard/Netherlands/00/95 (H7N3)	Ferrets	The vaccine virus replication was not detected in animal respiratory tissues; however, high levels of HAI antibodies were induced which cross-reacted with heterologous H7N9 virus; animals were protected from H7N3 and H7N9 wild-type viruses by reducing virus replication in URT and LRT; passively immunized ferrets were protected against lethal challenge with H7N9 virus, reduced weight loss and viral titers in URT	[14], [15]
A/17/California/66/395 (H2N2)	Ferrets	Single immunization elicited very high titers of homologous and heterologous HAI, MN and NAI antibodies; protected animals from homologous and heterologous challenge by reducing virus titers in URT and LRT and nasal turbinate tissue damage	[8]
A/17/Anhui/2013/61 (H7N9)	Ferrets	Both single and two-dose immunizations were highly immunogenic, prevented H7N9 wild-type virus replication in respiratory tissues and protected animals against severe bronchopneumonia	[9]

HAI – hemagglutination inhibition; HPAI – highly pathogenic avian influenza virus; LAIV – live attenuated influenza vaccine; LRT – lower respiratory tract; MN – microneutralization; NAI – neuraminidase inhibition; URT – upper respiratory tract.

**Table 4:** LAIV candidates recommended by WHO for the northern and southern hemisphere, used by SIIPL for manufacturing LAIV in 2011–2016.

Influenza season	Type/subtype	Recommended strain	Vaccine strain designation	Prepared by
2011 SH	H1N1	A/California/07/2009	A/17/California/2009/38	IEM
2011–2012 NH	H3N2	A/Perth/16/2009	A/17/Perth/09/87	IEM
2012 SH	B	B/Brisbane/60/2008	B/56/Brisbane/60/08	Nobilon

2012–2013 NH	H1N1	A/California/07/2009	A/17/California/2009/38	IEM
2013 SH	H3N2	A/Victoria/361/2011	A/17/Victoria/2011/89	IEM
	B	B/Wisconsin/1/2010	B/60/Wisconsin/1/2010	IEM

2013–2014 NH	H1N1	A/California/07/2009	A/17/California/2009/38	IEM
2014 SH	H3N2	A/Texas/50/2012	A/17/Texas/2012/30	IEM
2014–15 NH	B	B/Massachusetts/2/2012	B/60/Massachusetts/2012/10	IEM

2015 SH	H1N1	A/California/07/2009	A/17/California/2009/38	IEM
2015–16 NH	H3N2	A/Switzerland/9715293/2013	A/Switzerland/9715293/2013CDC–LV10A	CDC
	B	B/Phuket/3073/2013	B/60/Phuket/2013/26	IEM

2016 SH	H1N1	A/California/07/2009	A/17/California/2009/38	IEM
	H3N2	A/Hong Kong/4801/2014	A/17/Hong Kong/2014/8296	IEM
	B	B/Brisbane/60/2008	B/Texas/02/2013–CDC–LV8B	CDC

CDC – Centers for Disease Control and Prevention; IEM – Institute of Experimental Medicine; LAIV – live attenuated influenza vaccine; NH – northern hemisphere influenza season; SH – southern hemisphere influenza season; SIIPL – Serum Institute of India Pvt. Ltd.; WHO – World Health Organization.
